# Higher serum occludin after successful reperfusion Is associated with early neurological deterioration

**DOI:** 10.1111/cns.13830

**Published:** 2022-03-26

**Authors:** Weili Li, Shuhua Yuan, Xueqin Sui, Hetao Bian, Ming Wei, Zhiying Chen, Haitao Shao, Wenjuan Shi, Shuhai Shi, Xunming Ji

**Affiliations:** ^1^ Beijing Institute of Brain Disorders Laboratory of Brain Disorders Ministry of Science and Technology Collaborative Innovation Center for Brain Disorders Capital Medical University Beijing China; ^2^ Cerebrovascular Diseases Research Institute Xuanwu Hospital of Capital Medical University Beijing China; ^3^ 576398 Department of General Medicine Affiliated Hospital of Weifang Medical University Shandong Province China; ^4^ 117918 Department of Neurosurgery Tianjin Huanhu Hospital Tianjin China; ^5^ Department of Neurology Xuanwu Hospital of Capital Medical University Beijing China; ^6^ Department of Critical Care Medicine Beijing Shijitan Hospital Capital Medical University Beijing China; ^7^ Department of Neurosurgery Xuanwu Hospital of Capital Medical University Beijing China

**Keywords:** biomarker, blood‐brain barrier, early neurological deterioration, endovascular thrombectomy, occludin, stroke

## Abstract

**Aims:**

Early neurological deterioration (END) is an important factor that affects prognosis in patients with acute ischemic stroke. We explored the relationship between serum occludin levels after successful reperfusion and END in patients treated with endovascular thrombectomy (EVT).

**Methods:**

We prospectively enrolled 120 stroke patients who underwent EVT with successful reperfusion. Enzyme‐linked immunosorbent assay was used to detect the serum occludin levels on admission and within 1 h after successful reperfusion. Receiver operating characteristic curves (ROC) and regression analysis were used to compare the relationship between serum occludin and END after thrombectomy.

**Results:**

Among the 120 patients, 36 (30%) experienced END. The END group had higher serum occludin levels than the non‐END group after successful reperfusion [4.31 (3.71–5.38) vs 6.32 (5.88–6.99), *p* < 0.001]. The ROC curve showed that postoperative serum occludin levels had a significant prediction value for END (AUC: 0.86, *p* < 0.001). Regression analysis showed that serum occludin was an independent risk factor for END in EVT patients (adjusted odds ratio: 4.46, 95% confidence interval: 1.92–10.32; *p* < 0.001).

**Conclusions:**

The higher serum occludin levels were strongly related to END after successful reperfusion. Serum occludin may be an independent risk factor for END in EVT patients.

## Graphical abstract

This study makes a significant contribution because it provides novel evidence that the higher serum occludin levels after successful reperfusion could predict the risk of END in the next 24–48 h. It also indicates that blood–brain barrier damage during reperfusion may be an important risk factor for END in the future.

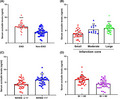

## 1 INTRODUCTION

Stroke is a hot topic: It is the number one disease threatening human health and is associated with high morbidity, disability, and strong recurrence. Moreover, acute ischemic stroke (AIS) is the most common type of stroke.^1^, ^2^ In the light of scientific advancements, endovascular thrombectomy (EVT) is recommended as a clinical guideline to treat AIS with large vessel occlusion.^3^ However, some patients undergoing EVT have a poor prognosis, and the early neurological deterioration (END) is an important factor that affects the 90‐day functional outcome.^4^ Therefore, understanding the potential mechanism of END may aid preventative decisions. The underlying mechanisms may include symptomatic intracranial hemorrhage (sICH), postoperative re‐occlusion, enlarged infarcts, thromboembolic events, and undetermined causes.^5^, ^6^, ^7^ Current clinical evaluations are mainly based on neurological function scores and brain imaging examinations, which also present key challenges. First, the accuracy of neurological function scores is poor. Second, imaging examinations require a long time and may not be recognized early on, despite the emergence of the disease. Therefore, there is an urgent need to develop novel detection methods. Although serum markers are widely studied, there is currently a lack of specific markers to this end.^8^

Occludin is a structural component of the tight junction protein of the blood‐brain barrier (BBB) and plays an important role in maintaining its integrity.^9^ Current studies have found that serum occludin is an important marker of BBB destruction in ischemic stroke and is closely related to ischemic stroke.^10^, ^11^ Our previous study found that serum occludin levels could be used to identify early hemorrhagic transformation of AIS.^12^ However, it remains unclear whether serum occludin was correlated with END for AIS patients who experienced EVT with successful reperfusion.

Herein, we aimed to identify the incidence of END in stroke patients with successful EVT and whether postoperative serum occludin levels were correlated with the occurrence of END.

## 2 METHODS

### 2.1 Study design

This research was carried out as a prospective observational study. The study was approved by the ethics committee of Xuanwu Hospital, and all patients provided signed informed consent.

For AIS patients undergoing EVT, blood samples will be collected on admission and within 1 h after successful reperfusion. Thereafter, enzyme‐linked immunosorbent assay (ELISA) was used to detect the occludin levels in the patients’ blood. We then collected the clinical data of the patients, including general baseline data, postoperative cerebral infarction volume at 24–48 h after onset, neurological function score (NIHSS), END within 24–48 h, and 90‐day clinical outcomes (Barthel index). Statistical methods were used to investigate the relationship between serum occludin and END, cerebral infarction volume, neurological score, and 90‐day clinical outcomes. In addition, we conducted stratification according to the infarction volume, NIHSS score, END, and Barthel Index to compare the differences between serum occludin levels among different groups.

### 2.2 Patient selection

We included 120 patients who underwent EVT with successful reperfusion for acute anterior circulation large vessel occlusion, treated in the emergency neurology department of Xuanwu Hospital from September 2019 to July 2021. All patients met the following indications for EVT: (1) previous stroke modified Rankin score (mRS) 0–1 points; (2) acute ischemic stroke caused by internal carotid artery or middle cerebral artery (MCA) M1 segment occlusion; (3) aged over 18 years; (4) National Institute of Health Stroke Scale (NIHSS) score ≥6 points; (5) Alberta Stroke Program early CT Score (ASPECTS) ≥6 points; (6) stroke occurred within 6 h; or within 6–24 h, but brain computed tomography perfusion imaging (CTP) showed obvious ischemic penumbra (CBF/CBV‐mismatch); (7) when the patient met the indications for intravenous rt‐PA thrombolysis, intravenous thrombolysis should be accepted and arranged EVT at the same time; and (8) when there was a contraindication to intravenous thrombolysis, EVT should be performed directly; (9) the patient and their family members signed an informed consent form. Exclusion criteria included (1) no postoperative blood samples; (2) inflammatory or infectious diseases, cancer, hemorrhagic disease, and severe renal and liver failure; (3) patients with missing important data; and (4) patients with failed recanalization.

### 2.3 Collection of clinical indicators

General baseline information included age, gender, past medical history, baseline NIHSS score, ASPECT score, time from onset to recanalization; intravenous thrombolysis (yes or no), the Trial of Org 10172 in acute stroke treatment (TOAST) classification, occluded vessel, and EVT strategies (mechanical thrombectomy combined with stenting, mechanical thrombectomy only). The prognostic evaluation data include infarct volume within 24–48 h of onset, 24‐h NIHSS score, incidence of END, and the Barthel index at 90 days. Successful reperfusion or recanalization was defined as a modified Thrombolysis in Cerebral Infarction (mTICI) score of 2b or 3.^13^

### 2.4 Image data

#### 2.4.1 Cerebral infarct volume

The baseline infarct volume was estimated by CT perfusion imaging. Magnetic resonance imaging diffusion‐weighted (MRI‐DWI) was used to detect cerebral infarct volume within 24–48 h after EVT. We adjusted the window width and layer thickness, traced the infarction area of each layer along the edge with the mouse, added the area of all layers, and multiplied the layer thickness to obtain the cerebral infarction volume.^14^ According to the size of the infarct volume, we defined the small (0–30 ml), medium (31–50 ml), and large (>50 ml) core infarct groups.^15^

### 2.5 Clinical score

#### 2.5.1 Neurological function score

The NIHSS score was used to evaluate the degree of neurological deficit, and the NIHSS score was defined as mild to moderate stroke if the NIHSS score is less than 17 points; the score was above 17 as severe stroke.^16^ It was divided into two groups. The NIHSS score ranged from 0 to 40 points and was assessed on site by two trained neurologists.

#### 2.5.2 90‐day prognosis score

The prognosis assessment was based on the 90‐day Barthel index as the evaluation standard and was assessed by on‐site assessment or telephone follow‐up. A Barthel index >80 was defined as a good prognosis, and ≤80 was defined as a poor prognosis.^17^

### 2.6 Early neurological function deterioration (END)

END was defined as the worsening of neurological dysfunction within 24 to 48 h, and the NIHSS score decreased by ≥4 points.^18^ The main causes of END included infarct expansion, symptomatic intracranial hemorrhage (sICH), re‐occlusion, thromboembolic events, and undetermined causes.^6^, ^19^, ^20^ Moreover, sICH was determined according to the ECASS‐III diagnostic criteria (intracranial hemorrhage confirmed by CT or MRI scan within 22–36 h with an increase in NIHSS score ≥ 4 points; intracranial hemorrhage is the main cause of neurological deterioration).^21^ Conversely, vascular re‐occlusion within 24–48 h was assessed by CTA (CT angiography) or MRA (magnetic resonance angiography). Intracranial hemorrhage was evaluated by non‐contrast computed tomography. Infarct expansion and thromboembolic events were evaluated by MRI‐DWI. If sICH, re‐occlusion, infarct expansion, and thromboembolic events were excluded, we defined END as undetermined causes.

### 2.7 Collection of blood samples and detection of serum occludin

Venous blood (2‐ml venous blood was extracted from the patient within 24 h of onset, placed at room temperature for 2 h, centrifuged, and placed in an EP tube and stored at −80°C until serum was detected). Centrifuge setting parameters: 3000 rpm, 145,000 g, 10 min. A commercially available ELISA kit (Occludin: USCN, China) was used to detect the serum occludin levels.

### 2.8 Statistical analysis

The data are presented as mean ± standard deviation or median (interquartile range, IQR). For continuous variables, normal distribution was assessed using the Kolmogorov–Smirnov test. Student's *t*‐test or ANOVA test was conducted for normal/Gaussian distribution data. If the data did not exhibit a normal distribution, we used a Mann–Whitney test. For categorical variables, we used a chi‐square test or Fisher's exact test. A correlation analysis was used to determine the correlation between serum occludin levels and cerebral infarction. The correlation coefficient r was used to determine the correlation strength. The value range of the correlation coefficient was generally between −1 and 1. The closer the absolute value is to 1, the stronger the linear relationship between variables, and the closer the absolute value is to 0, the weaker the linear relationship between variables. Values ≥0.8 indicated a strong degree of correlation, values ranging from 0.5 to 0.8 indicated a moderate degree of correlation, values ranging from 0.3 to 0.5 indicated a low degree of correlation, and values <0.3 indicated a very weak correlation and could be regarded as irrelevant. The receiver operating characteristic (ROC) curve was used to analyze the evaluation value of serum occludin in AIS. Multivariate regression analysis was used to judge the correlation between serum occludin and END and clinical prognosis at 90 days. The model was adjusted for age, gender, the baseline NIHSS score, ASPECT score, and TOAST classification and location of occlusion. The odds ratio (OR) and 95% confidence intervals (CI) were calculated. All data were analyzed using SPSS 23.0 (IBM Corporation, Armonk, NY, USA) software. *p* < 0.05 was considered as statistically significant.

## 3 RESULTS

### 3.1 Patients baseline characteristics

We screened 120 eligible patients from 148 patients receiving EVT, excluding 15 patients with recanalization failure, 8 patients with loss of follow‐up, and 5 patients with loss of blood samples. Among these 120 patients, there were 90 men and 30 women, with an average age of 63 years (Table 1).

**TABLE 1:** Demographic and clinical characteristics of admitted patients

Variable	Endovascular treatment (*n* = 120)
Age—y	63.0 ± 10.5
Male sex—*n* (%)	90 (75)
Atrial fibrillation—*n* (%)	45 (38)
Coronary artery disease—*n* (%)	33 (28)
Diabetes mellitus—*n* (%)	37 (31)
Hypertension—*n* (%)	80 (67)
Previous stroke—*n* (%)	35 (29)
Treatment with intravenous alteplase—*n* (%)	67 (56)
Median NIHSS score (IQR)	14 (12–16)
Median ASPECTS score (IQR)	9 (8–10)
Onset to puncture—min (IQR)	39 (28–50)
Onset to recanalization time—h (IQR)	6.3 (4.7–8.6)
Etiology of stroke—*n* (%)	
Large artery atherosclerosis	78 (65)
Cardioembolism	37 (31)
Other	5 (4)
Occluded vessel—*n* (%)	
Internal carotid artery	48 (40)
Middle cerebral artery	72 (60)
mTICI = 2b/3—*n* (%)	120 (100)
Endovascular treatment methods	
Mechanical thrombectomy only—*n* (%)	89 (74)
Stent implantation—*n* (%)	31 (26)

Note

Data were expressed as mean (SD), median (interquartile range), or number (%).

Abbreviations: ASPECTS, Alberta Stroke Program Early CT Score; IQR, interquartile range; mTICI, modified thrombolysis in cerebral infarction; NIHSS, National Institutes of Health Stroke Scale.

### 3.2 Serum occludin and END

**FIGURE 1:**
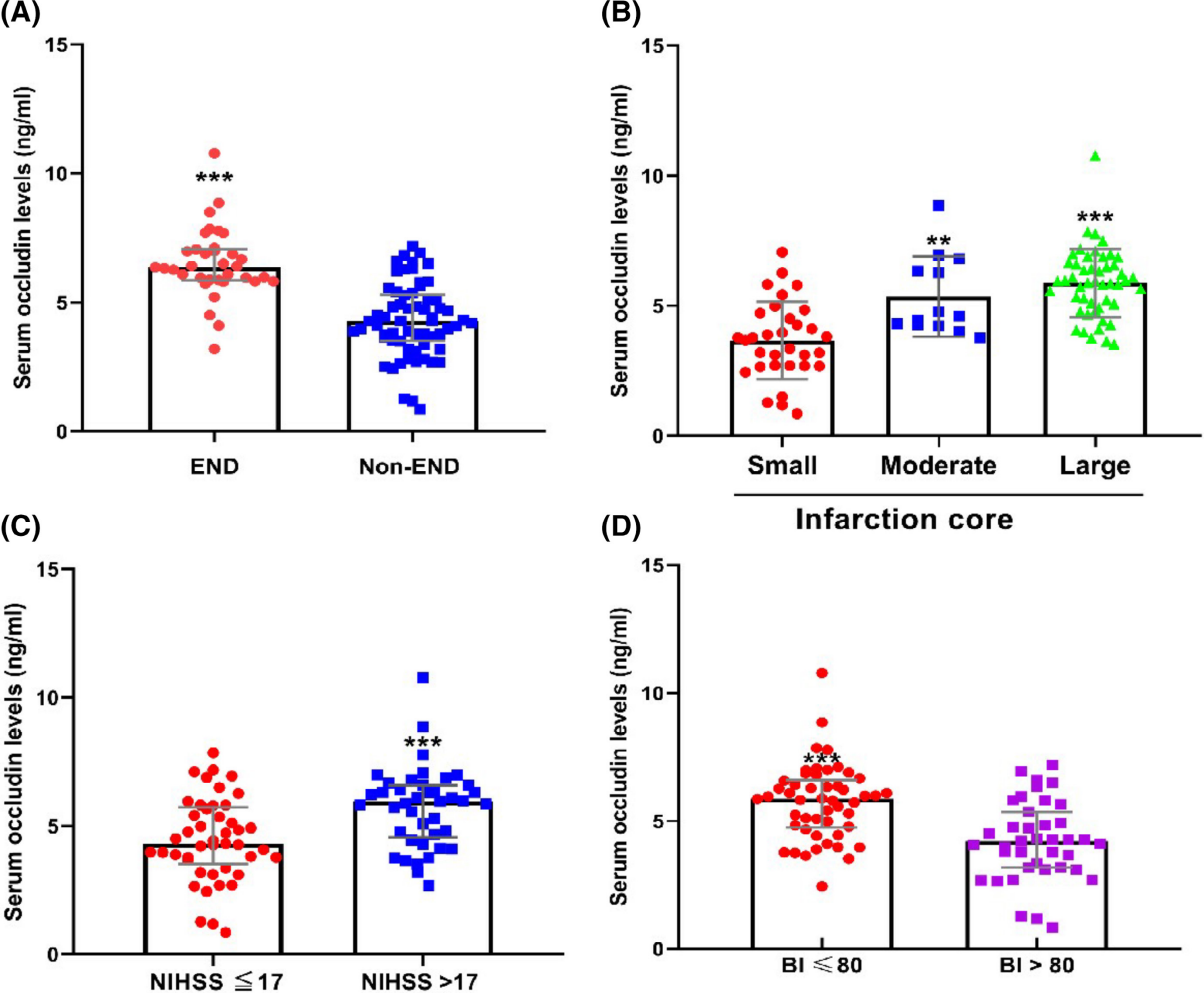
Serum occludin levels in different subgroups. (A) The serum occludin levels of the END group were significantly higher than those of the non‐END group (*p* < 0.001). (B) The larger the infarct core volume, the higher the serum occludin levels. The difference between the large infarct core group and the smaller infarct core group was statistically significant (*p* < 0.001), and the difference between the middle infarct core group and the smaller core infarction group was also statistically significant (*p* = 0.002). (C) The serum occludin levels in the NIHSS score >17 group were significantly higher than those in the NIHSS score ≦ 17 group; the difference was significant (*p* = 0.001). (D) The serum occludin levels of the poor prognosis group (Barthel index ≤ 80) were significantly higher than those of the good prognosis group (Barthel index > 80) (*p* < 0.001)

Among the 120 patients, 36 cases developed END (30%), including 13 cases of sICH (11%), 12 cases of re‐occlusion (10%), 3 cases of new stroke (2.5%), 5 cases of enlarged infarcts (4.4%), and 3 cases of unknown cause (2.5%). There were no significant differences in age, gender, previous medical history, baseline NIHSS score, ASPECT score, baseline infarct volume, and time from onset to recanalization between the END and non‐END groups. In addition, the baseline occludin levels had a rising tendency in END group than non‐END group [4.99 (3.20–5.88) vs 4.14 (3.03–5.16) ng/ml], although not statistically different (*p* = 0.081) (Table 2). However, we were surprised to find that the serum occludin levels after successful thrombectomy were significantly higher in the END group than the non‐END group [6.32 (5.88–6.99) vs 4.31 (3.71–5.38) ng/ml, *p* < 0.001] (Table 3 and Figure 1A).

**TABLE 2:** Comparison of baseline data of patients

Variable	END group (*n* = 36)	Non‐END group (*n* = 84)	*p* value
Age—y	67.7 ± 12.9	65.6 ± 8.1	0.281
Male sex—*n* (%)	25 (70)	65 (77)	0.630
Coronary artery disease—*n* (%)	12 (33)	21 (25)	0.608
Atrial fibrillation—*n* (%)	17 (48)	28 (33)	0.275
Diabetes mellitus—*n* (%)	13 (37)	24 (29)	0.585
Hypertension—*n* (%)	28 (77)	52 (62)	0.223
Previous stroke—*n* (%)	12 (33)	23 (27)	0.722
Treatment with intravenous alteplase—*n* (%)	23 (63)	44 (52)	0.487
Median NIHSS score (IQR)	14 (12–16)	13 (12–16)	0.592
Median ASPECTS score (IQR)	8 (7–9)	9 (8–10)	0.222
From onset to recanalization time—h (IQR)	6.5 (5.1–8.8)	6.2 (4.4–8.5)	0.472
Site of artery occlusion, *n* (%)			
Intracranial internal carotid artery	19 (52)	29 (35)	0.205
Middle cerebral artery	17 (48)	55 (65)	
Etiology of stroke—*n* (%)			
Non‐Cardioembolism^a^	20 (56)	63 (75)	0.058
Cardioembolism	16 (44)	21 (25)	
Stenting	7 (19)	24 (29)	0.460
Volume of infarct core^b^—ml (IQR)	16.8 (3.4–46.8)	14.9 (8.5–23.8)	0.634
Serum occludin at baseline (ng/ml)	4.99 (3.20–5.88)	4.14 (3.03–5.16)	0.081

Note

Data were expressed as mean (SD), median (interquartile range), or number (%).

Abbreviations: ASPECTS, Alberta Stroke Program Early CT Score; IQR, interquartile range; NIHSS, National Institutes of Health Stroke Scale.

^a^ Non‐cardioembolism includes large artery atherosclerosis type and other types.

^b^ Core infarct volume was estimated by CT perfusion imaging. For baseline CT perfusion imaging, thirteen patients were obtained in the END group; 45 were obtained in the non‐END group.

**TABLE 3:** Subgroup analysis of serum occludin levels

Subgroup	*N*	Occludin levels (ng/ml)	*p* value
END	120		
Yes	36	6.32 (5.88–6.99)	<0.001
No	84	4.31 (3.71–5.38)	
Infarct volume—ml (IQR)	120		
≦30	41	3.77 (2.68–4.72)	<0.05^a^
31–50	16	4.60 (4.22–6.69)	
>50	63	5.88 (4.91–6.57)	
NIHSS score at 24 h	120		
≦17	64	4.31 (3.51–5.72)	0.001
>17	56	5.96 (4.56–6.58)	
Barthel index at 90 days	120		
≦80	52	4.21 (3.18–5.35)	<0.001
81–100	68	5.87 (4.75–6.60)	

Abbreviations: END, early neurological deterioration; NIHSS, National Institute of Health Stroke Scale.

^a^ Serum occludin level of the three groups were different (*p* < 0.05); pairwise comparisons found that there were statistical differences between the small infarction group and the medium infarction group, and between the small infarction group and the large infarction group (*p* = 0.002, *p* < 0.001); However, there was no difference between the medium infarction group and the large infarction group (*p* = 0.168).

**FIGURE 2:**
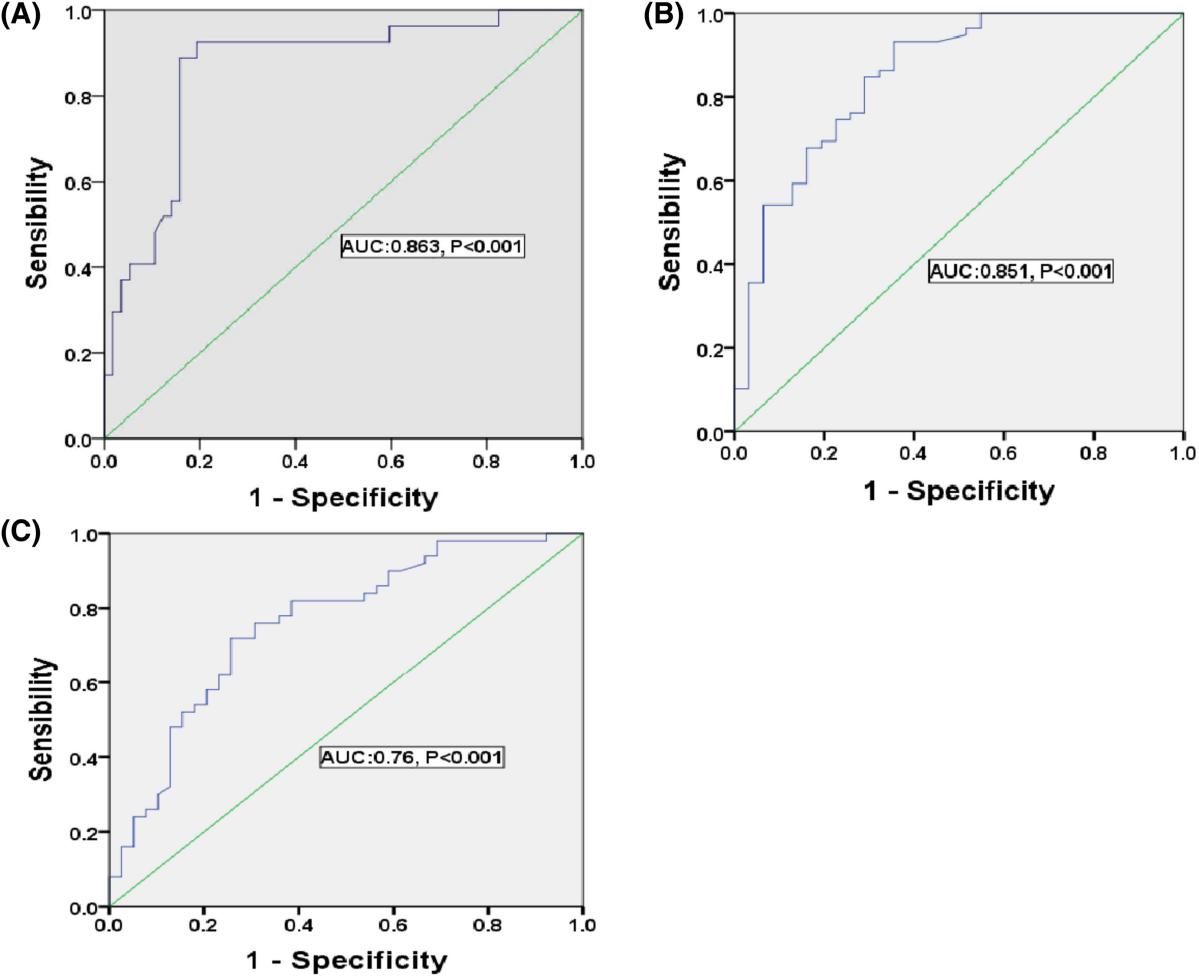
Receiver operating characteristic curve for serum occludin on predicting early neurological deterioration (A), infarct volume (B), and poor prognosis (C)

The ROC curve showed that the 24 h‐serum occludin levels had strong predictive value for END (AUC: 0.86, 95% CI: 0.78–0.95, *p* < 0.001). The best cut‐off value was 5.69 ng/ml, while the sensitivity and specificity were 93% and 81%, respectively (Figure 2A).

After adjusting for age, gender, NIHSS score, ASPECT score, stenting, site of artery occlusion, and etiology of stroke, the multivariate regression analysis found that serum occludin levels after thrombectomy were an independent risk factor for END in stroke patients who had undergone successful thrombectomy (adjusted OR = 4.46, 95% CI: 1.92–10.32, *p* < 0.001) (Table 4).

**TABLE 4:** Multivariate regression analysis of END

Variable	Effect variable^a^	Adjusted value (95% CI)	*p* value
24 h‐serum occludin level	Beta coefficient	4.46 (1.92–10.32)	<0.001
Occludin changes before and after EVT	Beta coefficient	2.22 (1.43–3.44)	<0.001
Age	Beta coefficient	1.10 (1.01–1.20)	0.038
Gender	Odds ratio	1.44 (0.25–8.27)	0.681
Baseline NIHSS score	Beta coefficient	0.78 (0.60–1.01)	0.061
Baseline ASPECT score	Beta coefficient	0.67 (0.39–1.13)	0.134
Site of artery occlusion	Odds ratio	3.70 (0.84–16.29)	0.084
Etiology of stroke	Odds ratio	6.70 (0.92–48.84)	0.061
Stenting	Odds ratio	1.20 (0.21–6.68)	0.839

Abbreviations: ASPECT, Alberta Stroke Program early CT; END, early neurological deterioration; EVT, endovascular thrombectomy; NIHSS, National Institute of Health Stroke Scale.

^a^ Values were adjusted for age, gender, NIHSS score, ASPECT score, stenting, site of artery occlusion, and etiology of stroke.

Moreover, the results showed that serum occludin changes before and after EVT in the END group were also significantly higher than that in the non‐END group [2.44 (0.89–3.35) vs 0.96 (0.62–1.59) ng/ml, adjusted OR = 2.22, 95% CI: 1.43–3.44, *p* < 0.001] (Table 4).

### 3.3 Serum occludin and infarct volume

The results demonstrated that there was a significant correlation between serum occludin levels and cerebral infarction volume after thrombectomy (r = 0.698, *p* < 0.001). Serum occludin levels in both the moderate and large infarct volume groups were significantly higher than those in the small infarct volume group [4.60 (4.22–6.69) vs 3.77 (2.68–4.72) ng/ml, *p* = 0.002; 5.88 (4.91–6.57) vs 3.77 (2.68–4.72) ng/ml, *p* < 0.001] (Table 3 and Figure 1B). Moreover, the ROC curve showed that serum occluding levels had a good predictive value in the medium‐large infarct core (AUC: 0.851, 95% CI: 0.764–0.938, *p* < 0.001), the best cut‐off value was 4.74 ng/ml, as seen in Figure 2B.

Preoperative quantitative penumbra data were obtained by CT perfusion image. We collected CTP data from 58 of the 120 patients. However, the results suggested that there was no correlation between serum occludin levels after EVT and the preoperative ischemic penumbra volume (r = −0.08, *p* = 0.618).

### 3.4 Serum occludin and NIHSS score

Serum occludin levels after thrombectomy were found to be significantly correlated with the NIHSS score within 24 h of a stroke (r = 0.52, *p* < 0.001). Moreover, the serum occludin levels were significantly higher in the NIHSS > 17 group than in the NIHSS ≤ 17 group [5.96 (4.56–6.58) vs 4.31 (3.51–5.72) ng/ml, *p* = 0.001] (Table 3 and Figure 1C).

### 3.5 Serum occludin and 90‐day prognosis

We found that 24‐h serum occludin levels correlated with Barthel index (r = 0.529, *p* < 0.001). In addition, the group with Barthel index ≤80 had higher serum occludin levels than the group with Barthel index >80 [5.87 (4.75–6.60) vs 4.21 (3.18–5.35) ng/ml, *p* < 0.001] (Table 3 and Figure 1D).

A ROC curve analysis was further performed, and results showed that the serum occludin levels after thrombectomy had a certain predictive value for poor prognosis at 90 days (AUC: 0.76, 95% CI: 0.658–0.861, *p* < 0.001); the best cut‐off value was 4.95 ng/ml (Figure 2C).

After adjusting for age, gender, NIHSS score, ASPECT score, stenting, site of artery occlusion, and etiology of stroke, a multivariate regression analysis revealed that 24‐h serum occludin levels >4.95 ng/ml were an independent risk factor for poor prognosis (Barthel index ≤ 80) for stroke patients who undergone successful thrombectomy (OR = 5.48, 95% CI: 1.36–22.08, *p* = 0.017), see Table S1.

## 4 DISCUSSION

This is the first study to explore the correlation between serum occludin levels and the occurrence of END after EVT with successful reperfusion. We confirmed that high serum occludin levels were closely related to END for stroke patients with successful EVT. In addition, postoperative serum occludin levels were correlated with the volume of cerebral infarction, 24‐h NIHSS score, and 90‐day functional outcome for patients who underwent EVT with successful reperfusion. Generally, END occurs 2–3 days after surgery, and it is difficult to identify it as early as possible through clinical evaluation or imaging methods.^4^ Previous studies suggested that END was a key factor leading to poor prognosis at 90 days. As serious complications of EVT, hemorrhage transformation, postoperative re‐occlusion, and thromboembolic events are common causes of END, with a high incidence, as assessed primarily by imaging and clinical symptoms in early stages.^22^, ^23^, ^24^ Interestingly, our study found that testing the serum occludin levels immediately after successful reperfusion could predict the risk of END in the next 24–48 h, which was clinically important. A similar study reported that BBB disruption was associated with increased mortality after EVT.^25^ The difference from this study was that BBB disruption was observed by CT imaging scan.

Previous studies indicated that AIS patients with increased serum occludin levels at baseline had a high risk of hemorrhage transformation, regardless of non‐reperfusion therapy or reperfusion therapy.^12^ In addition, several reports also showed the correlation between occludin degradation and ischemic reperfusion injury. Wang's study showed that MiR‐30a inhibitor restored the loss of occludin in microvessels of ischemic stroke rats and attenuate BBB breakdown and ischemic infarction, which conduced better outcome.^26^ Xu's study also showed that ischemic reperfusion injury significantly decreased those of the tight junction (TJ) proteins occludin and ZO‐1, and AIM2 knockdown effectively protected BBB integrity by promoting the expression of occludin and ZO‐1 proteins,^27^ and Izawa et al. suggested that inter‐endothelial claudin‐5 expression significantly decreased when β1‐integrin–collagen IV interactions were interrupted and subsequently extended the observations to the loss of ZO‐1 and of occludin at the inter‐endothelial interface, ultimately increasing BBB permeability.^28^ The above three studies demonstrated that the degradation of occludin protein caused the increase in BBB permeability with results of disease progression and complications. Our study was consistent with the above views. We agreed that serum occludin levels would increase when occludin was degraded in ischemic tissue, which can predict or correlate significantly with the occurrence of END after successful reperfusion. However, there are also different views. An interesting study found that TJs in the BBB promote edema formation and infarct size in stroke. Winkler et al. believed that Cldn3 and occludin could not only protect tight junctions and kept the BBB intact in stroke, but also promoted edema and infarction, as they described the ambivalent effects of sealing proteins.^29^ However, there is currently a lack of other evidence to support the interesting findings.

Previous animal studies have confirmed that elevated serum occludin levels could reflect the destruction of the BBB.^10^, ^11^ The interesting finding of this study is that there was no significant difference in BBB damage between the two groups before reperfusion. After successful reperfusion, the BBB damage in the END group was significantly heavier than that in the non‐END group. It may be that the reperfusion injury aggravated the BBB damage. In addition, it may be attributed to the damage of vascular endothelial cells caused by repeated thrombus removal, which increases the destruction of the BBB during EVT, and further damage to the BBB would lead to the production of inflammatory cytokines (MMP‐9/MMP‐2/IL‐6/IL1‐β/TNF‐α, etc.), which rapidly increase throughout the brain, and activates multiple injury mechanisms (i.e., oxidative stress, calcium overload, and apoptosis).^30^, ^31^, ^32^ Consequently, this creates a vicious circle that promotes the ischemic cascade reaction and aggravates brain tissue necrosis,^30^ resulting in END. Therefore, we speculate that BBB injury is closely related to the occurrence of END during reperfusion therapy. In other words, BBB damage caused by EVT may be the main mechanism for the occurrence of END. By detecting the postoperative serum occludin levels, we can judge the damage of the BBB, so as to predict the possibility of END in the future.

For the prediction of END after EVT, in addition to judging by serum markers, brain imaging assessment can also be used, such as early observation of cerebral edema by cranial CT, and intracranial contrast extravasation observed by cranial CT to judge BBB injury, which is helpful to predict the occurrence of END. In addition, cerebral hyperperfusion syndrome is a common type of END, which can be identified early by observing cerebral blood flow velocity through transcranial Doppler (TCD).^33^ Studies showed that postoperative cerebral blood flow velocity increased by more than 100% compared with that before surgery, suggesting cerebral hyperperfusion syndrome.^34^ We can also use postoperative CTP imaging to observe cerebral blood flow (CBF), cerebral blood volume (CBV), and mean transit time (MTT) to identify END.^33^, ^35^ In short, postoperative serum biomarker (occludin) combined with cranial imaging (CT/CTP/TCD) could significantly improve the detection rate of END, which will have important guiding value for clinicians.

We know that the neurological function score (NIHSS) 24 h after stroke is very important for estimating disease severity and prognosis.^36^, ^37^ Our study is the first to find that serum occludin levels are correlated with NIHSS scores after EVT. In addition, patients with higher NIHSS scores have higher serum occludin levels, which suggests that postoperative serum occludin levels could reflect the degree of neurological damage. Moreover, the detection of serum occludin is more objective, convenient, and concise compared with neurological function scores (NIHSS). For patients who do not cooperate well with early neurological function tests, it is difficult to undergo assessments of their condition through the NIHSS score. However, the detection of serum occludin may reflect the degree of neurological impairment and determine the degree of disease, reflecting its important clinical value as an auxiliary diagnostic tool. In the future, serum occludin combined with NIHSS score may better predict the disease severity and functional outcome of stroke patients after EVT.

### 4.1 Study limitations

There are some limitations which should be emphasized when explaining the results. Firstly, the relatively small sample size limits the power of our study. Secondly, some other clinical factors may be also correlated with the END, which needs to be further investigated. Thirdly, we did not include patients with failed recanalization, so it is not clear whether there is a correlation between serum occludin levels and vascular recanalization. Finally, individual patients were replaced by CT due to missing MRI scans, which led to differences in the infarct volume results. In the future, it will be necessary to add an analysis to clarify the correlation between END and serum occludin in the two groups of patients with successful or failed recanalization and also increase the sample size to confirm the reliability of the results.

## 5 CONCLUSIONS

In conclusion, there was a high incidence of END after successful reperfusion, and the high occludin levels were strongly related to END. It may be an independent risk factor for END for stroke patients who have undergone EVT with successful reperfusion. Postoperative serum occludin levels were also correlated with the volume of cerebral infarction, 24‐h NIHSS score, and 90‐day functional outcome after successful EVT.

## CONFLICT OF INTEREST

There are no conflicts among the authors.

## AUTHOR CONTRIBUTIONs

Weili Li, Xueqin Sui, and Xunming Ji involved in study concept and design. Shuhua Yuan and Xueqin Sui involved in patients enrollment and data collection. Weili Li, Shuhua Yuan, and Xueqin Sui involved in drafting the manuscript. Weili Li, Haitao Shao, Wenjuan Shi, and Shuhai Shi performed the experiments. Weili Li, Ming Wei, and Zhiying Chen involved in statistical analysis. All authors critical revised the manuscript. Xueqin Sui and Xunming Ji supervised the study.

## Supporting information

Table S1

## Data Availability

The data that support the findings of this study are available from the corresponding author upon reasonable request.
