# Supplementary material for: Higher serum occludin after successful reperfusion Is associated with early neurological deterioration
Source: CNS Neurosci Ther. 2022 Mar 26;28(7):999–1007. doi: 10.1111/cns.13830 (PMC9160448; doi:10.1111/cns.13830)
Supplement: Supplementary file 1 — Table S1 [file CNS-28-999-s001.docx]

**Supplementary material**

| **eTable 1.** Multivariate regression analysis of poor stroke prognosis (Barthel index ≤ 80) | | | |
| --- | --- | --- | --- |
| **Variable** | **Effect**  **Variable ^a^** | **Adjusted**  **Value (95% CI)** | **P Value** |
| Serum occludin levels | Beta coefficient | 5.48 (1.36-22.08) | 0.017 |
| ^a^ Values were adjusted for age, gender, NIHSS score, ASPECT score, stenting, site of artery occlusion, and etiology of stroke. | | | |
